# Severe Pediatric Ocular Surface Disease: Multimodal Approach and Treatment

**DOI:** 10.3390/pediatric18050134

**Published:** 2026-10-06

**Authors:** Laura Vilaverde, Bruno B. Ribeiro, Ricardo Parreira, Miguel Gomes, Pedro Menéres, Irene Barbosa

**Affiliations:** 1 Department of Ophthalmology, Unidade Local de Saúde de Santo António, 4099-001 Porto, Portugal; 2 Instituto de Ciências Biomédicas Abel Salazar, Universidade do Porto, 4050-313 Porto, Portugal

**Keywords:** ocular rosacea, ocular surface disease, blepharitis, intense pulsed light therapy, pediatric ophthalmology

## Abstract

Background/Objectives: Pediatric ocular surface disease associated with ocular rosacea, chronic blepharitis and meibomian gland dysfunction may result in significant corneal morbidity. In refractory cases, the limitations of conventional therapy have stimulated interest in adjunctive treatment modalities, including intense pulsed light (IPL) therapy. Methods: We report the case of a 10-year-old female patient with cutaneous rosacea, recurrent chalazia and chronic blepharitis who was referred for specialized ocular surface evaluation due to persistent ocular surface disease despite previous treatment. Results: At presentation, slit-lamp examination revealed diffuse keratitis, eyelid telangiectasia, inferior corneal neovascularization and corneal scarring. Despite the severity of ocular surface findings, no significant ocular complaints were documented and the OSDI-12 score was low. Ocular surface analysis demonstrated reduced non-invasive tear break-up time, incomplete blink rate, reduced lipid layer thickness and decreased tear meniscus height. Following optimization of conventional therapy, IPL treatment and adjunctive warming massage mask therapy, early improvement was observed in several ocular surface parameters, together with reduction of eyelid inflammation, improvement of keratitis and partial regression of corneal neovascularization. Subsequent longitudinal measurements showed variability. Conclusions: Pediatric ocular surface disease may present with severe objective findings despite minimal subjective symptoms, potentially delaying diagnosis and treatment. Careful evaluation is therefore essential in children with recurrent chalazia and chronic blepharitis. A multimodal therapeutic approach was associated with clinical and objective improvement in this patient; however, the contribution of individual treatment components cannot be established from a single case.

## 1. Introduction

Dry eye disease (DED) is a worldwide disease that affects up to 30% of the world’s population over 50 years of age, and conveys significant disability to both patients and financial cost to health systems [1]. It can be classified as evaporative or aqueous deficiency, despite most patients exhibiting mixed characteristics [1,2,3]. Risk factors include increasing age, female gender, meibomian gland dysfunction, and systemic medications (such as antihistamines, anticholinergics, and antidepressants) [2]. Although DED has traditionally been underrecognized in pediatric patients, recent studies have shown that ocular surface abnormalities and signs compatible with DED may be relatively common in children, reaching approximately 50% in some selected pediatric cohorts [4,5]. These findings highlight that pediatric ocular surface disease may be more prevalent than previously appreciated, although direct prevalence comparisons with adult populations should be interpreted cautiously because of differences in study populations and diagnostic criteria.

Currently, DED remains a challenging diagnosis, and its impact on patients’ quality of life has stimulated development of innovative diagnostic and treatment modalities. Thus, there is no single test to achieve a definite diagnosis of DED [3,4,5,6].

Intense pulsed light (IPL) is a broad-spectrum, non-coherent light-based therapy that has been increasingly used as an adjunctive treatment in meibomian gland dysfunction and evaporative ocular surface disease [7]. Treatment is delivered to the periocular skin using device-specific parameters selected according to patient characteristics such as skin phototype, with appropriate ocular protection throughout the procedure [8,9].

The therapeutic effects of IPL are thought to be multifactorial. Proposed mechanisms include thermal modification of altered meibum, reduction of periocular telangiectatic vessels, modulation of local inflammatory pathways and possible antimicrobial effects, which may collectively improve meibomian gland function and tear-film stability [7,8,9].

Therefore, we report a case of pediatric ocular surface disease associated with cutaneous rosacea, recurrent chalazia, chronic blepharitis and severe corneal involvement, managed with a multimodal therapeutic approach including conventional therapy, IPL and warming massage mask treatment. Pediatric ocular rosacea remains an underrecognized condition and delayed diagnosis may result in significant corneal morbidity [10].

## 2. Case Description

A ten-year-old female patient with recurrent chalazia and chronic blepharitis was referred for specialized ocular surface evaluation in September 2022. Past medical history was relevant for cutaneous rosacea. Previous ophthalmic history was notable for recurrent chalazia, for which several conventional treatments had been prescribed, including lid hygiene, warm compresses, topical lubricants, anti-inflammatory agents, antibiotics and omega-3 supplementation, with limited success. Poor adherence to the prescribed treatment regimen was also identified throughout follow-up.

**Figure 1:**
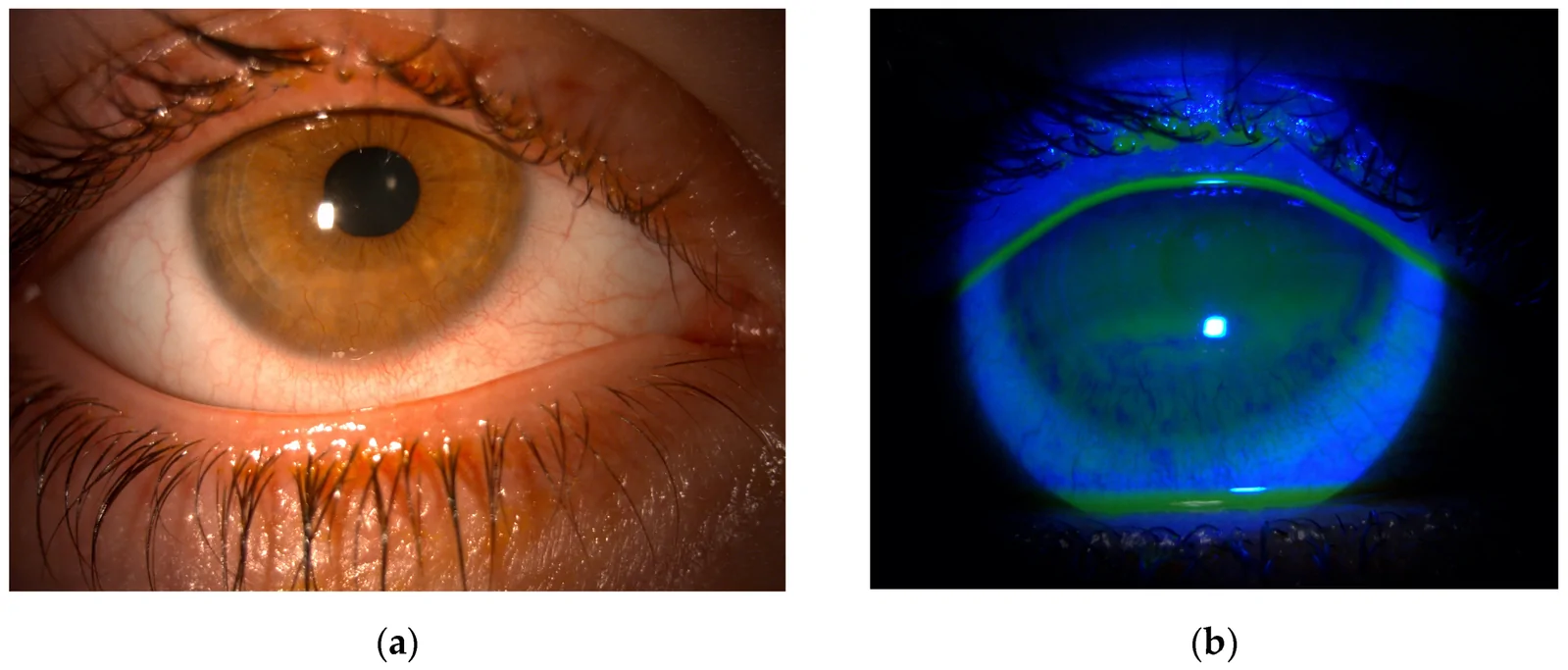
Eye before treatment. (**a**) Severe blepharitis, corneal neovascularization and scarring; (**b**) severe corneal fluorescein staining.

At presentation, best-corrected visual acuity was 20/25 in the right eye (OD) and 20/20 in the left eye (OS). Slit-lamp examination revealed chronic anterior and posterior blepharitis, diffuse keratitis, conjunctival hyperemia, upper and lower eyelid telangiectasia, inferior corneal neovascularization and corneal scarring (Figure 1). The clinical picture was considered to represent severe pediatric ocular surface disease associated with ocular rosacea, chronic blepharitis and meibomian gland dysfunction, rather than primary dry eye disease. No formal diagnostic criteria were applied to establish DED as an independent diagnosis; the tear-film abnormalities identified on ocular surface assessment were interpreted in the context of the underlying inflammatory ocular surface disease. Despite the severity of ocular surface findings, no significant ocular complaints were documented at presentation.

Automated ocular surface analysis was performed using the IDRA system (SBM Sistemi, Rivalta di Torino, Italy), according to the standardized device protocol, with automatic processing of the acquired measurements. The reported values correspond to the device-generated outputs for each parameter. Schirmer’s test and tear film osmolarity were also performed at the first consultation. NIBUT was reported as the automatic average time generated by the device, while blink analysis, lipid layer thickness and tear meniscus height were recorded according to the corresponding standardized IDRA outputs. Ocular surface symptoms were assessed using the Ocular Surface Disease Index-12 (OSDI-12). Baseline multimodal ocular surface analysis revealed reduced non-invasive tear break-up time (NIBUT) (5.3 s in OD and 7.6 s in OS), incomplete blink rate (50.0% in OD and 56.0% in OS), reduced lipid layer thickness (55 nm in both eyes) and reduced tear meniscus height (0.16 mm in OD and 0.14 mm in OS). Tear osmolarity was 316 mOsm/L in OD and 286 mOsm/L in OS. Schirmer’s test measured 25 mm in both eyes, while the OSDI-12 score was 7 points.

Conventional therapy was reinforced, including ocular lubrication, lid hygiene and measures aimed at reducing ocular surface stress. Particular attention was given to treatment adherence, avoidance of eye rubbing, appropriate blinking behavior and reduction of prolonged screen exposure. Clinical improvement was observed, with reduction of eyelid secretions and inflammatory signs. Given the persistence of clinically significant eyelid and ocular surface inflammation, meibomian gland dysfunction and corneal involvement despite optimization of conventional therapy, IPL was considered as an individualized adjunctive treatment. IPL was performed using the E>Eye device (E-Swin, Houdan, France), according to the standard device protocol. Protective eye shields were used and conductive gel was applied to the treatment area. Five flashes were delivered per eye under the lower eyelid, progressing from the medial to the lateral side. According to the manufacturer’s technical specifications, the device uses intense regulated pulsed light (IRPL), with a pulse duration of 170 ms and an emission wavelength range of 580–1625 nm. An energy setting of 13.0 J/cm^2^ was used, corresponding to the lowest available setting and selected according to the patient’s skin phototype. These pulse characteristics were predefined by the device, and no specific adjustment was made based on the patient’s age. A four-session treatment cycle was performed.

Follow-up examinations showed progressive improvement of blepharitis and ocular surface inflammation. Additional treatment with a warming and massage eye mask (Activa^®^, SBM Sistemi, Italy) was subsequently prescribed. The device was applied for four weekly sessions followed by one additional session two months later. Due to discomfort associated with the facial support of the device, further sessions were discontinued.

**Figure 2:**
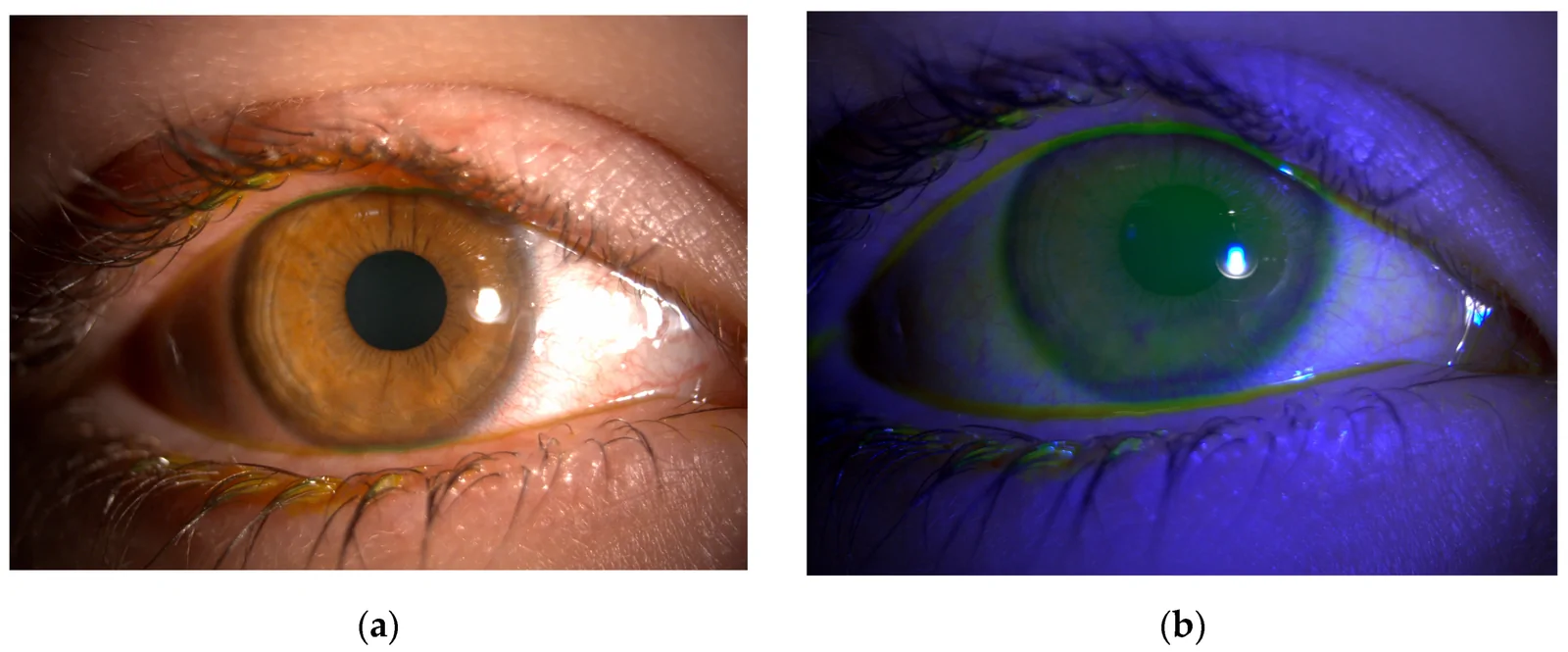
Eye after treatment. (**a**) Marked improvement of blepharitis and partial regression of corneal neovascularization; (**b**) marked improvement of corneal fluorescein staining.

**Figure 3:**
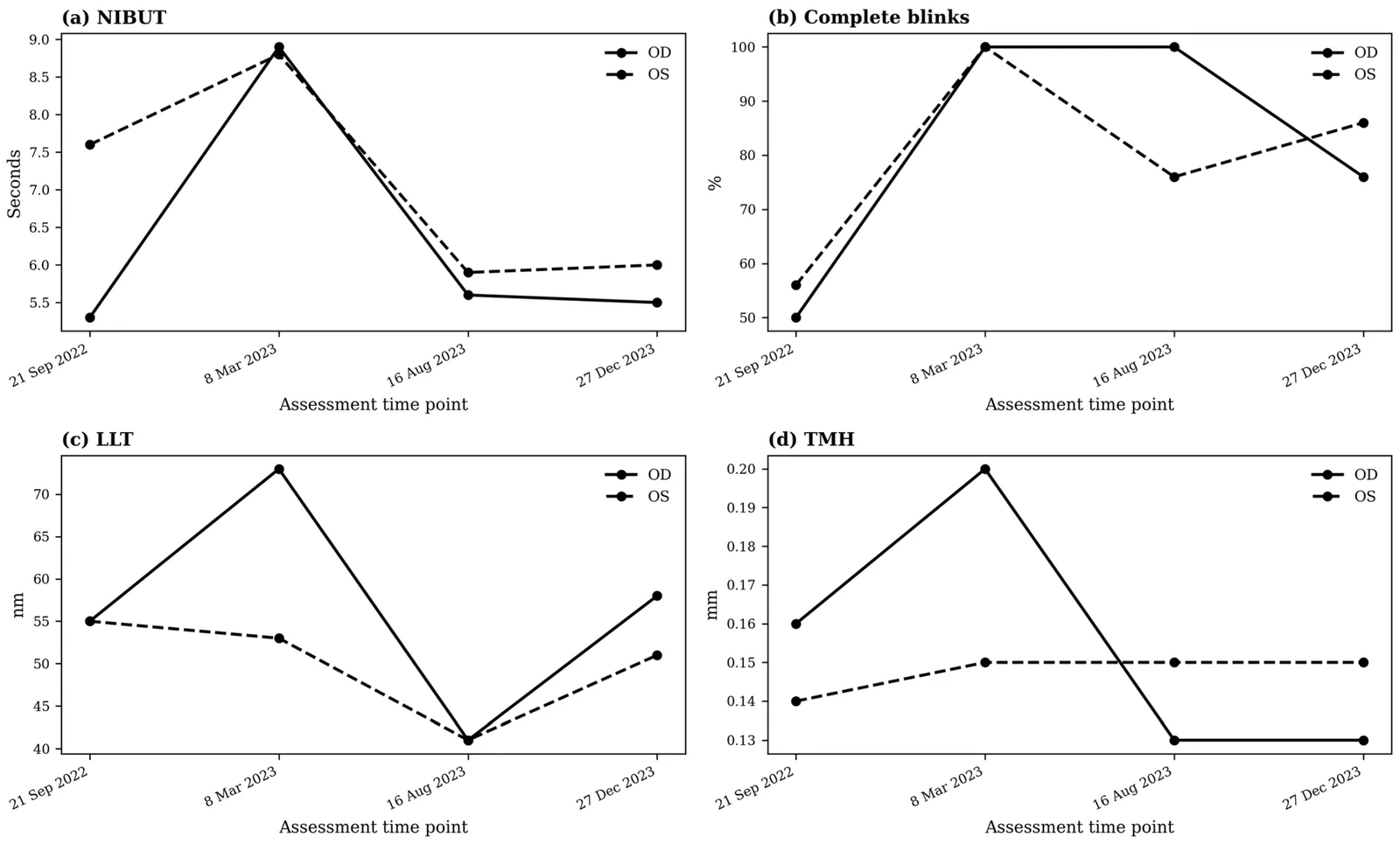
Longitudinal changes in objective ocular surface parameters during follow-up. (**a**) Non-invasive tear break-up time (NIBUT); (**b**) complete blink rate; (**c**) lipid layer thickness (LLT); and (**d**) tear meniscus height (TMH). Values are shown separately for the right eye (OD) and left eye (OS) at baseline (21 September 2022) and at follow-up assessments on 8 March, 16 August, and 27 December 2023.

At the early post-treatment assessment on 8 March 2023, approximately one month after completion of the first IPL cycle, NIBUT was 8.9 s in OD and 8.8 s in OS, the proportion of complete blinks was 100% in both eyes, lipid layer thickness was 73 nm in OD and 53 nm in OS, and tear meniscus height was 0.20 mm in OD and 0.15 mm in OS. At the approximately six-month follow-up on 16 August 2023, NIBUT was 5.6 s in OD and 5.9 s in OS, complete blinks were 100% in OD and 76% in OS, lipid layer thickness was 41 nm in both eyes, and tear meniscus height was 0.13 mm in OD and 0.15 mm in OS (Table 1). Tear osmolarity was not reassessed at follow-up. Slit-lamp examination demonstrated reduction of eyelid inflammation, improvement of keratitis and partial regression of corneal neovascularization (Figure 2). The longitudinal evolution of NIBUT, complete blink rate, lipid layer thickness and tear meniscus height is illustrated in Figure 3.

At the most recent follow-up visit, the patient remained clinically stable, without ocular complaints and with unchanged visual acuity. Occasional chalazia continued to occur, in the context of suboptimal adherence to the prescribed treatment regimen. Ongoing maintenance therapy and periodic follow-up were recommended.

## 3. Discussion

Our patient presented with severe pediatric ocular surface disease associated with ocular rosacea, chronic blepharitis and meibomian gland dysfunction, characterized by recurrent chalazia and significant corneal involvement. In this case, DED was not considered the primary diagnosis; rather, the tear-film abnormalities were interpreted in the context of the underlying inflammatory ocular surface disease. The keratitis, inferior corneal neovascularization and corneal scarring were considered manifestations of the inflammatory corneal involvement associated with pediatric ocular rosacea/blepharokeratoconjunctivitis, rather than consequences of DED per se. Pediatric ocular rosacea may manifest with chronic blepharitis, recurrent chalazia, meibomian gland dysfunction and progressive corneal involvement, potentially resulting in significant morbidity when diagnosis and treatment are delayed [10].

A particularly relevant aspect of this case was the discrepancy between subjective symptoms and objective disease severity. Despite diffuse keratitis, inferior corneal neovascularization and corneal scarring, no significant ocular complaints were documented at presentation, and the OSDI-12 score was low. This may represent an important diagnostic challenge in pediatric patients, who may underreport symptoms or adapt to chronic ocular surface discomfort. This observation is consistent with previous reports demonstrating a poor correlation between subjective symptoms and objective ocular surface findings in pediatric populations, with many children presenting clinically significant ocular surface abnormalities despite minimal symptoms [5]. Therefore, careful slit-lamp examination remains essential in children presenting with recurrent chalazia and chronic blepharitis, even in the absence of significant complaints.

Another important challenge was treatment adherence. Compliance with conventional therapy had been inconsistent before referral and remained suboptimal throughout follow-up. This is particularly relevant in pediatric patients, as treatment often requires multiple daily interventions, including ocular lubrication, lid hygiene, warm compresses and behavioral measures such as reducing prolonged screen exposure, improving blink patterns and avoiding eye rubbing. Frequent eye rubbing in children should not be overlooked, as it may reflect underlying ocular surface irritation and may contribute to corneal epithelial damage, ocular surface inflammation and meibomian gland dysfunction [11]. Therefore, children presenting with recurrent eye rubbing should undergo careful ocular surface evaluation, and avoidance of this behavior should be specifically reinforced.

Parental involvement is also particularly important in the management of chronic ocular surface disease in children. Parents and caregivers play a central role in treatment implementation, recognition of abnormal ocular behaviors and reinforcement of preventive measures. Clear communication regarding the chronic and potentially recurrent nature of the disease, its possible corneal complications and the rationale for treatment may facilitate parental engagement and improve adherence, particularly when significant objective disease is present despite few subjective complaints [12]. In our patient, repeated counseling of both the patient and her parents was an important component of long-term management.

Initial management focused on optimizing conventional therapy, including preservative-free ocular lubricants, lid hygiene and anti-inflammatory treatment. Artificial tears containing trehalose, hyaluronic acid, carbomer and natural phospholipids were selected due to their complementary effects on tear film stability and ocular surface protection [13,14]. However, given the persistence of meibomian gland dysfunction and significant ocular surface abnormalities, additional treatment strategies were considered.

IPL has been increasingly investigated as an adjunctive treatment for meibomian gland dysfunction and evaporative ocular surface disease. Studies in adult populations have reported improvements in tear-film stability, meibomian gland function, eyelid margin inflammation and ocular surface symptoms following IPL treatment [7,8,9,15,16,17,18]. Proposed mechanisms include thermal liquefaction of abnormal meibum, reduction of periocular telangiectatic vessels, modulation of inflammatory mediators and possible antimicrobial effects, all of which may contribute to improved tear-film stability and reduction of ocular surface inflammation [7,8,9,15,17,18,19].

IPL is generally considered well tolerated when appropriate treatment parameters and ocular protection are used. Reported adverse effects are usually mild and transient and may include periocular discomfort, erythema, edema, skin irritation and pigmentary changes. Less commonly, thermal skin injury may occur. Because treatment is delivered in the periocular region, adequate eye protection is essential to minimize the risk of ocular injury [15]. In the present case, no treatment-related adverse events were documented.

Most evidence supporting IPL derives from adult populations, while pediatric-specific evidence remains limited. In the present case, IPL was considered on an individualized basis as an adjunctive treatment because clinically significant eyelid and ocular surface inflammation, meibomian gland dysfunction and corneal involvement persisted despite conventional therapy. The patient’s age was taken into account, but the treatment parameters were selected according to the device protocol and skin phototype rather than age.

Following the multimodal treatment approach, early improvement was observed in several objective ocular surface parameters, although subsequent measurements showed variability over time. Clinical improvement was also documented, including reduction of blepharitis, keratitis and corneal neovascularization. However, because conventional therapy, behavioral measures, IPL and warming/massage therapy were used in combination, the individual contribution of IPL cannot be determined. Although complete resolution was not achieved, disease stabilization and reduction of inflammatory signs were documented during follow-up.

The persistence of occasional chalazia likely reflects the chronic and recurrent nature of the underlying condition and may also have been influenced by incomplete adherence to maintenance therapy. Long-term follow-up remains essential to prevent recurrence, monitor corneal complications and preserve visual function.

This case report has several limitations. First, as a single-patient observation, the findings cannot be generalized to the broader pediatric population. Second, the intervention was multimodal, including optimization of conventional therapy, behavioral measures, IPL and adjunctive warming/massage therapy; therefore, the individual contribution of IPL to the observed clinical and objective improvement cannot be determined. Third, not all ocular surface parameters were available longitudinally, as tear osmolarity was not reassessed after treatment. In addition, changes in tests with known intra-individual variability, such as Schirmer testing, should be interpreted cautiously. Finally, evidence regarding the use of IPL in pediatric patients remains limited, and larger prospective studies with standardized treatment and assessment protocols are required to establish its safety and efficacy in this population.

## 4. Conclusions

Pediatric ocular surface disease may present with significant objective findings despite minimal subjective symptoms. In children with recurrent chalazia and chronic blepharitis, careful ocular surface evaluation is essential, as delayed diagnosis and treatment may lead to corneal scarring, neovascularization and long-term visual impairment.

This case highlights the importance of a multimodal and individualized approach, combining conventional therapy, behavioral measures, attention to treatment adherence and, in selected refractory cases, IPL therapy. In this patient, the multimodal therapeutic approach was associated with clinical and objective improvement; however, the contribution of any individual treatment component, including IPL, cannot be established from a single case. Education of both children and their caregivers is particularly important, including counseling on avoidance of eye rubbing, recognition of abnormal ocular behaviors and adherence to long-term treatment. Although complete resolution may not always be achieved, early intervention, parental engagement and long-term follow-up are essential to control inflammation, prevent recurrence and preserve visual function.

## Figures and Tables

**Table 1:** Clinical timeline, interventions, and objective ocular surface findings.

Time Point	Intervention/Clinical Event	Main Clinical Findings	Objective Ocular Surface Parameters
21 Sep 2022—Baseline	Initial specialized ocular surface assessment; conventional therapy reinforced	Recurrent chalazia; chronic anterior and posterior blepharitis; diffuse keratitis; conjunctival hyperemia; eyelid telangiectasia; inferior corneal neovascularization and corneal scarring	BCVA: 20/25 OD, 20/20 OS; NIBUT: 5.3/7.6 s; complete blinks: 50%/56%; LLT: 55/55 nm; TMH: 0.16/0.14 mm; tear osmolarity: 316/286 mOsm/L; Schirmer: 25/25 mm; OSDI-12: 7
18 Nov 2022–2 Feb 2023	Four-session IPL treatment cycle	Persistent eyelid and ocular surface inflammation and corneal involvement despite conventional treatment; IPL sessions documented without incident	-
8 Mar 2023—Early post-treatment assessment	Assessment after completion of the first IPL cycle; adjunctive warming/massage therapy subsequently initiated	Post-IPL clinical reassessment	NIBUT: 8.9/8.8 s; complete blinks: 100%/100%; LLT: 73/53 nm; TMH: 0.20/0.15 mm
16 Aug 2023—Approximately 6-month follow-up	Follow-up after IPL and adjunctive treatment	Reduced inflammatory signs, with persistent inferior corneal involvement	NIBUT: 5.6/5.9 s; complete blinks: 100%/76%; LLT: 41/41 nm; TMH: 0.13/0.15 mm
27 Dec 2023—Later follow-up	Maintenance treatment and longitudinal follow-up	Continued clinical surveillance	NIBUT: 5.5/6.0 s; complete blinks: 76%/86%; LLT: 58/51 nm; TMH: 0.13/0.15 mm

## Data Availability

The original contributions presented in this study are included in the article. Further inquiries can be directed to the corresponding author.

## Abbreviations

The following abbreviations are used in this manuscript:

DED	Dry eye disease
IPL	Intense pulsed light
NIBUT	Non-invasive tear break-up time
OD	Right eye
OS	Left eye
OSDI-12	Ocular Surface Disease Index 12 score
